# Quantitative imaging analysis in hypertrophic cardiomyopathy: phenotypic differentiation to prognostic stratification

**DOI:** 10.1093/ehjimp/qyag162

**Published:** 2026-09-12

**Authors:** S Pradeep Kundur, A Rai, A Kandala, A Malik, S Sivalokanathan, F Jamal, S Jha, A Owens

**Affiliations:** Faculty of Life Sciences and Medicine, King’s College London, Guy’s Campus, London SE1 1UL, UK; Department of Internal Medicine, University of Pennsylvania, Philadelphia, PA, USA; Honorary Clinical Researcher, Department of Medicine, Imperial College London, UK; University College London, London, UK; Faculty of Life Sciences and Medicine, King’s College London, Guy’s Campus, London SE1 1UL, UK; Icahn School of Medicine, Mount Sinai Fuster Heart Hospital, New York, NY 10025, USA; Department of Radiology, Hospital of the University of Pennsylvania, Philadelphia, PA, USA; Department of Radiology, Hospital of the University of Pennsylvania, Philadelphia, PA, USA; Department of Cardiology, University of Pennsylvania, Philadelphia, PA, USA

**Keywords:** hypertrophic cardiomyopathy, radiomics, cardiac imaging, machine learning, artificial intelligence, sudden cardiac death

## Abstract

Hypertrophic cardiomyopathy (HCM) is the most common inherited cardiac disorder and remains associated with sudden cardiac death (SCD), heart failure, and diagnostic uncertainty due to overlap with other causes of left ventricular hypertrophy, including hypertensive heart disease and cardiac amyloidosis. This review aimed to evaluate the evolving applications of radiomics in the context of related quantitative imaging techniques. A review was conducted of studies applying radiomics, texture analysis, machine learning, and deep learning to cardiac imaging in HCM. The evidence base derives predominantly from CMR, with a smaller and more recent body of work in echocardiography and CT. Current evidence suggests that radiomics can extract quantitative features of myocardial texture, shape, and signal heterogeneity beyond visual assessment. These approaches have shown promise in differentiating HCM from phenocopies, identifying myocardial fibrosis without contrast administration, and improving the prediction of adverse outcomes, including SCD and heart failure. Several studies reported incremental value over conventional imaging markers such as wall thickness, global T1 values, and binary late gadolinium enhancement. However, the literature remains limited by retrospective study design, small cohorts, heterogeneity in imaging acquisition and segmentation, and insufficient external validation. Radiomics has emerged as a promising adjunct to conventional cardiac imaging in HCM, with the potential to be adapted into a clinical decision support tool. Its future clinical role will depend on methodological standardization and robust external validation.

## Graphical Abstract

**Graphical Abstract:**
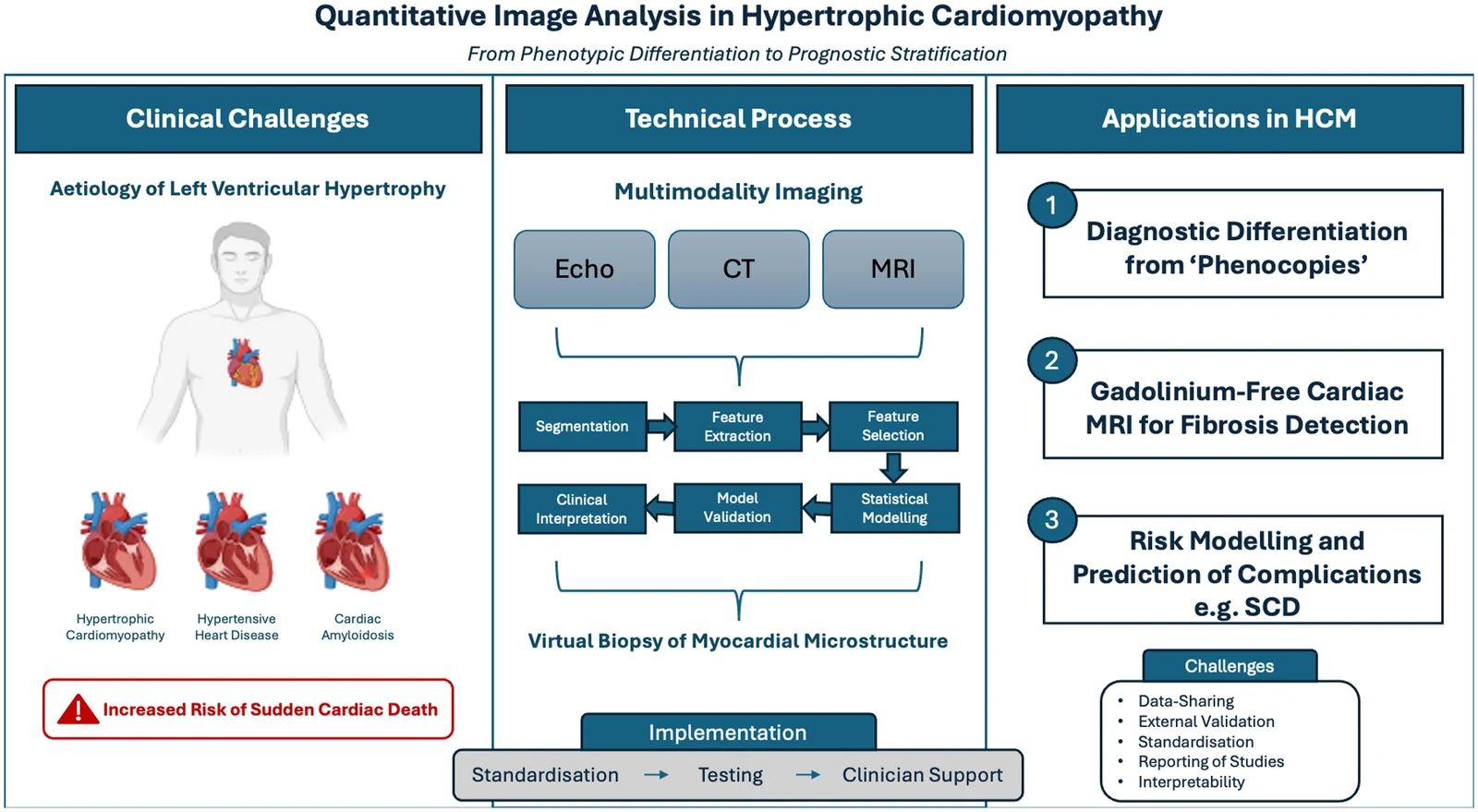

## Introduction

### Background

Hypertrophic cardiomyopathy (HCM) is the most prevalent inherited cardiac disease worldwide, affecting approximately 1 in 200 to 500 adults.^1^ Despite its prevalence, HCM is frequently underdiagnosed and remains the leading cause of sudden cardiac death (SCD) in young adults in the USA.^2–4^

### Definition and pathology

Current consensus from the European Society of Cardiology (ESC), American Heart Association (AHA), and American College of Cardiology (ACC) guidelines defines HCM as unexplained left ventricular hypertrophy (LVH) with echocardiography or cardiovascular magnetic resonance (CMR) showing a maximal end-diastolic wall thickness of ≥15 mm within the left ventricle,^5–7^ except in relatives of diagnosed patients or individuals with pathogenic variants for whom thresholds of 13–14 mm may suffice. Histologically, HCM is characterized by cardiomyocyte hypertrophy, myofibrillar disarray, and interstitial fibrosis, which underpins diastolic dysfunction and arrhythmogenic risk.^6^

### Current diagnostic pathways and limitations

Diagnosis of HCM is multimodal, integrating clinical assessment, imaging, and genetic testing.^7^ Transthoracic echocardiography remains the primary tool for detecting LVH and left ventricular outflow tract obstruction, whereas CMR is the reference standard for tissue characterization and morphology.^8^ Late gadolinium enhancement (LGE) quantifies focal myocardial fibrosis, a surrogate of arrhythmogenic risk,^9^ while genetic testing supports diagnosis and family screening.^10^

Despite these tools, important diagnostic and prognostic gaps remain. Hypertensive heart disease (HHD), cardiac amyloidosis (CA), and athlete's heart can mimic HCM^11^ yet require different management, making differentiation essential. Histology with biopsy remains the definitive method of characterization, highlighting the need for non-invasive alternatives.

### The role of SCD risk

Current guidelines prioritize the prevention of SCD in HCM management, recognizing fibrosis as an incremental marker of arrhythmic risk, although its role in implantable cardioverter defibrillator (ICD) decision-making, which remains the most effective strategy to mitigate the risk of malignant arrhythmias,^5,12^ differs across guidelines. ACC/AHA considers extensive LGE (>15% LV mass) a Class IIb indication for ICD implantation,^13^ whereas ESC guidelines recommend shared decision-making in intermediate-risk individuals.^12^ Despite its widespread use, LGE lacks a strong negative predictive value. Although contemporary risk-prediction strategies have reduced SCD rates,^14^ residual risk persists. Functional markers such as myocardial strain detect early mechanical dysfunction but not tissue composition. Likewise, binary LGE and maximal wall thickness do not capture the diffuse microstructural abnormalities that contribute to arrhythmogenesis, reinforcing the need for complementary structural imaging biomarkers.

### The scope for radiomics

Current diagnostic frameworks rely on limited descriptors such as wall thickness, ejection fraction, and binary LGE. Radiomics addresses this by extracting quantitative features capturing myocardial texture, shape, and signal heterogeneity beyond visual assessment. Although the evidence base remains weighted towards CMR, where myocardial tissue characterization is most mature, this review synthesizes radiomics applications across cardiac imaging,^15^ incorporating the emerging but still limited literature in echocardiography and CT. By contrasting engineered radiomics with traditional analysis and data-driven deep learning (DL), we evaluate its capacity to bridge diagnostic gaps, reduce the burden of SCD, and overcome the standardization barriers requisite for clinical translation.

## Methods

This structured review was conducted in accordance with the PRISMA-ScR guidelines (*Figure 1*). A comprehensive literature search was performed in PubMed/MEDLINE, Embase, and Scopus from database inception to September 2025. The search strategy combined MeSH and free-text keywords across three domains: clinical condition, computational methodology, and imaging modalities. Studies were screened using predefined inclusion criteria, including peer-reviewed original studies applying radiomics, texture analysis, or machine learning to cardiac imaging in HCM with reported diagnostic or prognostic performance metrics. Reference lists of relevant articles were screened to identify additional eligible studies. Two reviewers independently screened studies and extracted data, resolving discrepancies by consensus. Extracted data included study characteristics, sample size, imaging modality, feature extraction methods, classification approaches, and key diagnostic or prognostic outcomes. A description of the included studies can be found in *Tables 1–3*.

**Figure 1:**
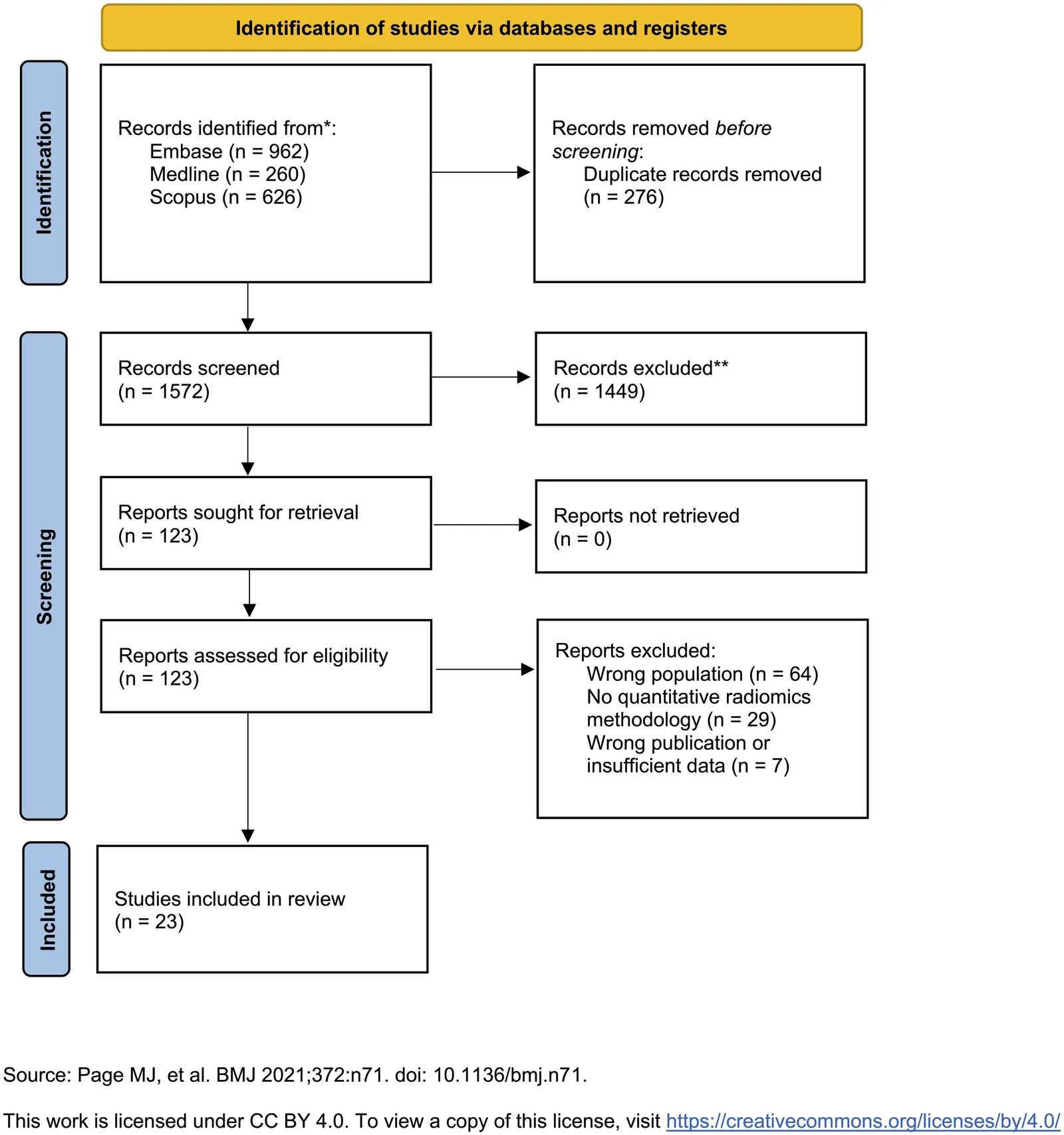
PRISMA ScR flowchart. Records were identified through searches of Embase, MEDLINE, and Scopus. Following removal of duplicate records, titles and abstracts were screened, with eligible full-text articles assessed against the predefined inclusion and exclusion criteria. Reasons for full-text exclusion included incorrect study population, absence of quantitative radiomics methodology, inappropriate publication type, and insufficient data. A total of 23 studies met the eligibility criteria and were included in the final systematic review.

**Table 1:** Characteristics of studies assessing HCM phenocopies

Study ID (First Author, Year)	Scanner brand/model	Region of interest (ROI)	Acquisition parameters	Segmentation method	Segmentation tool/software	Segmentation performed by	Radiomics features extracted	Best-Performing Feature(s)	Feature Selection Method	AI / ML Approach	Features Selected (Final Model)
**Moon *et al*., 2025^16^**	GE Vivid series, Philips EPIQ, multiple vendors	Entire LV myocardium (auto-segmented on TTE PLAX, PSAX mid, A4C, A2C at ED & ES)	Frame rate >45 fps; harmonic imaging; ED & ES extraction	Automated AI-based LV segmentation with cardiologist adjudication	AI pipeline (USfeat.ai; Ontact Health, Korea) + PyRadiomics	Expert cardiologists reviewed segmentation	19 839 features (texture, harmonization-driven texture, shape, thickness %Δ ED–ES)	Run entropy (GLRLM), Run-length non-uniformity, % change in thickness, % change in shape	Two-step: Boruta → XGBoost importance ranking	LightGBM (gradient boosting)	70 (LVH vs. N), 82 (HCM vs. CA/HHD), 83 (CA vs. HCM/HHD), 6 (HHD vs. HCM/CA)
**Zhang *et al*., 2024^17^**	Philips Ingenia 3T; Siemens Verio 3T; GE Discovery MR750 3T	Entire LV myocardium on cine bSSFP + LGE PSIR	Cine: 25 phases; slice 8 mm SAX, 5 mm LAX; LGE: 10 min post 0.2 mmol/kg Gd-DTPA	Auto cine segmentation + manual correction; manual LGE contours	cvi42 v5.11.2; PyRadiomics v3.0	Experienced operators (>5 yrs)	1409 features (semantic, first-order, texture, wavelet, LBP, etc.)	Cine + LGE radscores; top cine & LGE features	ICC >0.8 → Spearman (ρ<0.7) → multivariate regression weighting	Multivariate logistic regression	20 cine + 20 LGE features (cine, LGE, combined radscores)
**Zhang *et al*., 2023^18^**	Multi-vendor (Siemens, GE, Philips, Canon)	LV, RV, MYO (cine ED & ES)	Slice 5–8 mm; gap 5 mm; resolution 1.37–1.68 mm^2^/pixel; 28–40 phases/cycle	Manual delineation (expert annotators)	Public ACDC + M&Ms datasets	Clinicians at dataset centres, ICC 0.79–0.98	567 features per ROI (first-order, shape, texture, LOG, wavelet)	LeastAxisLength, Kurtosis, Sphericity, Flatness, Skewness	Multi-method: 10 selection algorithms → Permutation Feature Importance	Random Forest (best) vs. KNN, LR, XGB, SVM, NB, MLP, DT, EL	11 (7 MYO, 4 LV; mainly shape & first-order)
**Liu *et al*., 2023^19^**	Philips Ingenia 3T	LV myocardium + papillary muscles	Slice 6–8 mm; TR 3.0 ms; TE 1.4 ms; FA 45°; 30 phases; resolution 0.8–1.2 mm	Manual segmentation	ITK-SNAP v3.8.0 + cvi42 v5.13	2 cardiologists + senior reviewer	2632 total (1316 MYO, 1316 PM; wavelet, LoG, gradient)	MYO GLCM correlation, PM shape diameter, GLSZM ZoneEntropy	LASSO → Boruta ranking	Support Vector Machine	6 MYO, 6 PM (3 + 3 combined)
**Jiang *et al*., 2022^20^**	Siemens MAGNETOM Skyra 3T	LV myocardium on 4-chamber cine (end-systole)	SSFP cine; standard parameters; subgroup with LGE	Manual ROI on 4-chamber LAX	MaZda v4.6.0	2 radiologists (3 & 6 yrs exp)	275 (histogram, GLCM, GLRLM, gradient, wavelet)	Gray Level Non-Uniformity (GLRLM)	ICC >0.75 → LASSO	SVM (best), RF, NB, KNN, LR, ANN	19 (LASSO-selected); top = GLevNonU
**Linardos *et al*., 2022^21^**	Multi-vendor (Siemens, Philips, Canon)	LV, RV, MYO (ED & ES)	Resolution 0.8–1.8 mm; slice 5–10 mm	Manual segmentation (expert)	ACDC + M&Ms dataset annotations	Expert annotators; consensus rules	— (Deep learning only)	—	—	3D ResNet18 CNN (DL only)	—
**Xu *et al*., 2022^22^**	GE Vivid E9/E95, Philips EPIQ7C	IVS (A4C echo videos)	Frame rate >45 fps; harmonic imaging	Automated DL segmentation (ResUNet)	Custom PyTorch; MATLAB post-processing	Expert echo reviewer (training contours)	1150 total (static 46 + time/frequency domain 25)	Temporal/frequency-domain features (TD/FD)	*t*-test filter → Pointwise Gated Boltzmann Machine (PGBM)	SVM	Automatically selected by PGBM + *t*-test
**Izquierdo *et al*., 2021^23^**	Siemens Avanto 1.5T	LV myocardium + cavity (ED & ES)	TR/TE 3.2/1.5 ms; voxel 1.4 × 1.4 × 8 mm; 25 phases	Semi-automatic contouring	Circle CVI42	Expert cardiologist	420 (shape, first-order, GLCM, GLRLM, GLSZM, GLDM, NGTDM)	Texture (GLRLM, GLSZM) for HCM/LVNC; shape for DCM	Correlation filtering + SelectKBest (ANOVA)	SVM, RF, Logistic Regression	∼30–40 per model; top 10 per class
**Wang *et al*., 2021^24^**	Siemens MAGNETOM Trio Tim 3T	LV myocardium (LGE images)	PSIR; 0.2 mmol/kg Gd-DTPA	Manual segmentation	QMass 8.1 (Medis)	2 blinded radiologists	157 (first-order, shape, GLCM, LBP, etc.)	LBP (19) + Moment (1)	CoxNet + RF; bootstrapping + 10-fold CV	Cox regression; RF survival	2 final (LBP 19, Moment 1)
**Antonopoulos *et al*., 2021^25^**	Philips Ingenia 1.5T	Basal short-axis (native T1 MOLLI)	FOV 320 × 320 mm; slice 10 mm; TR/TE 2.3/1.08 ms; matrix 160 × 160	Manual endo/epi contouring (fat/blood excluded)	3D Slicer + SlicerRadiomics	Operators trained by a Level 3 CMR expert	850 (shape, first-order, GLCM, GLDM, GLRLM, GLSZM, NGTDM, wavelets)	LLH_glcm_Imc1, HLH_glszm_LAHGE, LLL_glcm_MCC, etc.	CV < 10% → correlation <0.8 → RF ranking	Multinomial logistic regression; RF	6 final stable features
**Yu *et al*., 2021^26^**	GE Vivid E9/7, Philips IE33/EPIQ7C	IVS (A4C, ED frame)	A4C; frame rate >45 fps	Manual segmentation	In-house MATLAB scripts	Experienced echocardiographer	77 (17 first-order + 60 GLCM)	EtBrt (entropy of brightness), Std, CoV	ANOVA + *t*-test/KW + ICC >0.5	SVM (RBF kernel)	EtBrt, Std, CoV
**Wang *et al*., 2020^27^**	Siemens MAGNETOM Skyra 3T	LV myocardium (native T1 MOLLI)	Cine bSSFP: TR 3.4 ms; TE 1.3 ms; FA 50°; slice 8 mm. MOLLI: TR 740 ms; TE 1.06 ms; voxel 2.4 × 1.8 mm	Manual segmentation (endo + epi)	QMass v8.1 (Medis)	Experienced radiologists (>2000 CMRs/yr)	157 (first-order, GLCM, GLRLM, LBP, etc.)	LBP-7, LBP-37, Run Percentage-2	PCA for reduction	SVM (linear)	3–5 dominant features (LBP-7, LBP-37, RP.2)
**Neisius *et al*., 2019^28^**	Siemens (T1 mapping)	LV myocardium (native T1 maps)	Multislice; full myocardium (no LGE)	Manual segmentation	In-house/standard mapping tools	Experienced radiologists	Texture descriptors (first-order, spatially independent/dependent)	6 best texture features	Sequential feature selection	SVM	6 final
**Schofield *et al*., 2019^29^**	Siemens Avanto 1.5T	Mid-ventricular short-axis myocardium (ED cine)	Voxel 2 × 2 × 8 mm; TR 39.6 ms; TE 1.12 ms; FA 55°	Manual ROI (single slice)	TexRAD (Cambridge, UK)	Operators supervised by >10 yrs exp	6 texture features (mean, SD, skewness, kurtosis, entropy, MPP)	Mean, SD, entropy, MPP; skewness, kurtosis	No ML; ICC>0.8 → statistical testing	ROC of single features	6 single features (SSF=3 mm)

Summary of studies applying radiomics and deep learning to distinguish hypertrophic cardiomyopathy (HCM) from phenocopies such as hypertensive heart disease (HHD), cardiac amyloidosis (CA), and other left ventricular hypertrophy (LVH) entities. Reported parameters include scanner model, region of interest (ROI), image acquisition, segmentation methods, extracted radiomic features, feature selection, and artificial intelligence (AI) or machine learning (ML) models used.

AI, artificial intelligence; ANN, artificial neural network; A4C, apical 4-chamber; bSSFP, balanced steady-state free precession; CA, cardiac amyloidosis; CNN, convolutional neural network; CV, cross-validation; DCM, dilated cardiomyopathy; ED, end-diastolic; ES, end-systolic; FA, flip angle; FOV, field of view; GLCM, gray-level co-occurrence matrix; GLDM, gray-level dependence matrix; GLRLM, gray-level run-length matrix; GLSZM, gray-level size zone matrix; HCM, hypertrophic cardiomyopathy; HHD, hypertensive heart disease; ICC, intraclass correlation coefficient; IVS, interventricular septum; LBP, local binary pattern; LGE, late gadolinium enhancement; LR, logistic regression; LV, left ventricle; LVH, left ventricular hypertrophy; ML, machine learning; MOLLI, modified Look-Locker inversion recovery; MPP, mean positive pixels; MYO, myocardium; NB, naïve Bayes; NGTDM, neighbourhood gray-tone difference matrix; PCA, principal component analysis; PSIR, phase-sensitive inversion recovery; RF, random forest; ROI, region of interest; SAX, short axis; SD, standard deviation; SVM, support vector machine; TTE, transthoracic echocardiography; XGB, XGBoost. Boruta, Boruta (random-forest–based) feature-selection algorithm; PGBM, probabilistic gradient boosting machine; TabPFN, tabular prior-data fitted network.

**Table 2:** Characteristics of studies predicting fibrosis

Study ID (first author, year)	Scanner brand/model	Region of interest	Acquisition parameters	Segmentation method	Segmentation tool/software	Segmentation performed by	Radiomics features extracted	Best-performing feature(s)	Feature selection method	AI / ML approach	Features selected (final model)
**Fahmy *et al*., 2022^30^**	Philips Achieva (*n* = 424); GE Signa Genesis (*n* = 11), Signa Excite (*n* = 127); Siemens Sonata (*n* = 5), Avanto (*n* = 62), Symphony (*n* = 1), Verio (*n* = 3), NA (*n* = 126); external: Philips Achieva	Entire LV myocardium from cine (short-axis stack, end-diastolic frame); scar quantified by manual thresholding	TR 2.5–3.6 ms; TE 1.1–1.7 ms; flip 39–60°; pixel 0.6–1.4 mm; slice 8–10 mm; gap 8–10 mm; 17–30 phases	Automatic LV border detection with manual correction	CVI42 v5.10 (Circle CVI)	Experienced operators (3–7 yrs CMR)	PyRadiomics v3.0.1; 14 shape + 93 texture (GLCM, GLRLM, LBP, histogram) × 9 filters (gradient, log, square, sqrt, exp, 4× wavelets); **total 944**	Shape + GLCM/GLRLM (varied by fold)	LASSO regression	Logistic regression (L1; balanced classes)	10 radiomics (rad-only model); 20 (DL + radiomics)
**Pu *et al*., 2023^31^**	GE Signa Excite HD 1.5T; Siemens MAGNETOM Avanto 1.5T	Entire LV myocardium on cine (short-axis, ED); features from MWT slice and full LV	Slice 8 mm; gap 2 mm; TR 3.5/2.64 ms; TE 1.5/1.11 ms; flip 45°/56°; FOV 360 × 360 / 340 × 276 mm^2^; temporal res ∼47–49 ms	Manual LV myocardium segmentation (ED)	ITK-SNAP v3.8.0	Radiologist A (4 yrs); repeatability vs. Radiologist B (5 yrs)	PyRadiomics v3.0.1; **1130**(MWT slice) & **1688** (entire LV): first-order, 2D/3D shape, 75 texture, 1023/1581 high-order	R2 model top 5 (mostly high-order / filtered)	Boruta after ICC >0.85	XGBoost (Extreme Gradient Boosting)	Top 100 → ∼5–10 key predictors (R2: 5 top); integrated model fused MWT, LVMI, GLS with radiomics
**Mancio *et al*., 2022** ^32^	Siemens, Philips, GE (1.5T)	Entire LV myocardium at ED from cine bSSFP; auto DL segmentation with manual correction	Standard cine bSSFP (∼25 phases/cycle; vendor-specific)	Automatic DL contours + manual adjustment	Cvi 42 v5.10	Experienced operators (J.M. 6 yrs; I.C. 4 yrs)	PyRadiomics; **2613** (first-order, shape, GLCM, GLDM, GLRLM, GLSZM, NGTDM; + gradient, LoG, wavelet, LBP)	7-feature signature: P90; GLSZM LAHGLE; GLCM MCC; GLDM dependency entropy; NGTDM complexity; GLRLM short-run emphasis (↓ in LGE+); shape mesh volume	XGBoost importance with 10-fold stratified CV	XGBoost (radiomics + wall thickness + wall thickening)	7 radiomics + per-segment wall thickness & thickening
**Neisius *et al*., 2020^33^**	Philips Achieva 1.5T, 32-ch coil	Entire LV myocardium on native T1 STONE (manual); LGE with CNN + manual correction	Native T1: slice-interleaved STONE, 5 SAX, free-breathing 90 s; LGE: 3D PSIR ∼15 min post 0.1 mmol/kg gadobenate	Manual endo/epi on T1; CNN-based LGE seg. with manual correction	MedIACare (custom); CNN for LGE	Readers U.N. (12 yrs), S.K. (8 yrs); reproducibility tested	**152** texture (histogram, run-length, co-occurrence, LBP)	5: LRLGE-45°, Homogeneity-4, LBP-1, LBP-16, LBP-22	ICC >0.75 → sequential forward selection → RUSBoost DTE ranking	Decision Tree Ensemble (RUSBoost)	5 final (LRLGE-45°, Homogeneity-4, LBP-1, LBP-16, LBP-22)
**Qin *et al*., 2021^34^**	Siemens SOMATOM Force (dual-source CT)	Myocardium segmented into AHA 17 segments on CCTA; ROI = segment + whole LV	Standard contrast-enhanced CCTA; tube voltage 80–100 kV; slice thk not fully specified	Automatic AHA 17-segment segmentation with manual adjustment	Frontier VB10 Radiomics (Siemens)	2 radiologists (5 & 10 yrs), blinded	**1691** (shape, first-order, GLCM, GLRLM, GLSZM, GLDM, NGTDM, wavelet/filtered)	Top 3: SumEntropy, Maximum (first-order), ClusterProminence (GLCM) after wavelet	Test–retest ICC >0.8 → mutual information → RF importance	ANN (MF presence); Random Forest (Rad-sig & MF ≥ 15%)	28 (Rad-sig) for MF ≥ 15%; top-ranked: SumEntropy, Maximum, ClusterProminence

Summary of studies employing radiomics and deep learning to quantify or predict myocardial fibrosis in HCM. Reported data include scanner specifications, ROI, image acquisition and segmentation methods, extracted radiomic features, feature selection strategies, and AI or ML models developed for fibrosis prediction.

AHA, American Heart Association; LoG, Laplacian of Gaussian; LV, left ventricle; RUSBoost, random undersampling boosting.

**Table 3:** Characteristics of SCD studies

Study ID (First author, Year)	Scanner brand/model	Region of interest (ROI)	Acquisition parameters	Segmentation method	Segmentation tool/software	Segmentation performed by	Radiomics features extracted	Best-performing feature(s)	Feature selection method	AI / ML approach	Features selected (final model)
**Durmaz *et al*., 2024^35^**	Siemens MAGNETOM Aera 1.5T	Hyperenhancing LV myocardium on LGE, propagated to cine images	Slice 8 mm; gap 2 mm; in-plane res. ∼1.5 × 1.5 mm; LGE 10–15 min after 0.2 mmol/kg gadolinium	Manual segmentation	3D Slicer v4.11	Two radiologists (5 & 8 yrs exp), adjudicated by a senior	1612 total (806 cine + 806 LGE): first-order, shape, GLCM, GLRLM, GLSZM, GLDZM, wavelet	18 selected (11 cine, 7 LGE); top: GLCM entropy, GLSZM gray-level non-uniformity, wavelet	LASSO regression (α = 0.005)	TabPFN (Transformer-based meta-learning classifier)	18 total features
**Zhang *et al*., 2024** ^36^	GE Discovery MR750 3T; Siemens MAGNETOM Verio 3T; Philips Ingenia 3T	Entire LV myocardium at end-diastole (cine bSSFP); radiomics from whole myocardium for outcome prediction	Standard cine bSSFP; slice thickness and TR/TE in Supplementary data online, *Table S1*; voxel resampled to 1 × 1 × 1 mm^3^	Automatic LV endo/epi segmentation (cine ED) + manual LGE contours	cvi42 v5.11.2 (Circle CVI)	Two operators (12 & 10 yrs exp); reproducibility vs. senior cardiologist (15 yrs)	PyRadiomics v3.0; 1408 features (first-order, shape, texture, wavelet, LoG); ICC >0.8	5 radiomics combined into Rad-score	ICC >0.8 → Spearman filtering → LASSO Cox regression	Cox proportional hazards regression (clinical + MRI predictors)	5 radiomics (Rad-score components)
**Fahmy *et al*., 2024^37^**	Philips & GE (1.5T)	Scar tissue maps from LGE; LV endo/epi contours manually drawn; radiomics from myocardium, including scar + normal	Standard LGE: 0.2 mmol/kg Gd-DTPA; 10–20 min post-contrast; 2D segmented IR/PSIR; TI adjusted to null myocardium	Manual myocardial contouring (LV endo/epi) + manual LGE thresholding	QMass v7.4 (Medis)	Experienced readers (blinded; 2 centres)	PyRadiomics v3.0.1; 14 shape + 930 texture; 9 filters (gradient, log, square, sqrt, exp, 4× wavelets); 944 features/slice → 1888/patient	NGTDM coarseness (scar heterogeneity)	PCA (variance-based, outcome-blinded)	Logistic regression (weighted for imbalance)	3 principal radiomics (PrinRads)
**Wang *et al*., 2024** ^38^	Philips Ingenia 3.0T (dStream torso coil)	Entire LV myocardium (endo/epi) on native T1 MOLLI (basal, mid, apical)	MOLLI: TR 2.3 ms; TE 1.0 ms; pixel 1.3 × 1.3 mm; slice 7 mm; FOV 300 × 300 mm. Cine bSSFP: TR 2.8 ms; TE 1.4 ms; pixel 1.2 × 1.2 mm; LGE PSIR: TR 6.1 ms; TE 3.0 ms; voxel 1.6 × 1.9 mm; slice 7 mm	Manual segmentation (endo + epi, basal/mid/apical)	3D Slicer v4.11.20210226	4 readers (3–4 yrs) checked by a senior (10 yrs)	PyRadiomics v3.0; 150+ (first-order, shape, GLCM, GLRLM, GLSZM, NGTDM, GLDM; wavelet, LoG, LBP)	Top 10: LoG_firstorder_Median, wavelet-L_firstorder_90th, glcm_ClusterProminence, glcm_MCC, glcm_Correlation, glrlm_RunVariance, glrlm_RunLengthNonUniformity, shape_MinorAxisLength, shape_Sphericity	Feature importance via PyCaret (permutation) → Extra Trees ranking	Extra Trees Classifier (radiomics); ResNet32 CNN (deep learning)	10 radiomics (ML); auto-learned DL features

Summary of radiomics and deep learning studies predicting sudden cardiac death or arrhythmic risk in hypertrophic cardiomyopathy (HCM). Reported parameters include scanner characteristics, region of interest (ROI), image acquisition and segmentation methods, extracted radiomic features, feature selection strategies, and artificial intelligence (AI) or machine learning (ML) models used for outcome prediction.

CNN, convolutional neural network; DL, deep learning; FOV, field of view; GLCM, gray-level co-occurrence matrix; GLDM, gray-level dependence matrix; GLRLM, gray-level run-length matrix; GLSZM, gray-level size-zone matrix; LBP, local binary pattern; LoG, Laplacian of Gaussian; LV, left ventricle; MOLLI, modified Look-Locker inversion recovery; NGTDM, neighbourhood gray-tone difference matrix; PCA, principal component analysis; PSIR, phase-sensitive inversion recovery.

## Radiomics: concepts and pipeline

### Image acquisition

The radiomics workflow begins with image acquisition. Consistency in acquisition parameters is essential to minimize technical variability. Reviews emphasize that image acquisition parameters (e.g. contrast, voxel size, reconstruction) remain the most influential sources of variance in radiomics, and that quality checklists should ensure strict standardization before features are extracted.^39^ Pre-processing improves feature comparability.

### Segmentation

Segmentation defines regions of interest for feature extraction, typically the hypertrophied myocardium or fibrotic regions. While manual segmentation is widely used, it introduces inter-observer variability and limits scalability.^39^ Automated deep learning approaches, particularly U-Net architectures, provide improved reproducibility and efficiency,^40^ achieving near-expert performance.^41–43^ However, segmentation in HCM remains challenging due to asymmetric hypertrophy and complex ventricular geometry, necessitating robust validation for downstream analysis.

### Feature selection and extraction

Radiomic features include first-order statistics (mean, variance, entropy) and higher-order texture features from gray-level matrices.^39^ PyRadiomics is commonly used for feature extraction. Given the high dimensionality, feature selection helps prevent overfitting.^44^ Dimensionality reduction techniques, such as Principal Component Analysis (PCA), can be applied to transform the dataset by mathematically combining correlated features into a smaller set of artificial components. While effective at reducing data size, PCA sacrifices clinical interpretability, as the resulting components cannot be mapped back to specific biological textures. This restricts analysis to the most robust and meaningful descriptors, retaining only features with a high intraclass correlation coefficient (ICC) across repeated scans.^45^

Conversely, feature selection algorithms retain the original, interpretable features by isolating the most predictive subset. Common techniques include filter methods, such as minimum redundancy maximum relevance (mRMR) and embedded methods like the least absolute shrinkage and selection operator (LASSO), which applies an L1 regularization penalty to shrink the coefficients of uninformative features to zero. Modern radiomics pipelines strongly favour feature selection over pure dimensionality reduction to maintain biological transparency. Yet recently, Bayesian feature selection is gaining traction for its utility in estimating feature inclusion and quantifying uncertainty.^46^

### Statistical models

Following feature selection, models are applied to classify aetiologies or predict outcomes. While logistic regression and support vector machines remain common, recent studies increasingly favour ensemble classifiers. Notably, Extreme Gradient Boosting (XGBoost) utilizes gradient-boosted decision trees to manage complex, non-linear feature interactions, frequently outperforming traditional classifiers. To counteract the inherent opacity of these advanced models, modern workflows integrate SHapley Additive exPlanations (SHAP). Grounded in cooperative game theory, SHAP quantifies the exact contribution of each radiomic feature to a patient's specific prediction,^47^ evolving complex computations into transparent clinical decision-support tools.

Concurrently, deep learning frameworks such as Convolutional Neural Networks (CNNs) can extract spatial texture directly from images,^48^ while U-Net architectures streamline automated boundary segmentation. Model efficacy is rigorously evaluated using conventional metrics, such as the area under the receiver operating characteristic curve (AUC), sensitivity, and specificity, alongside calibration and decision curve analyses.^49^ A schematic framework of these processes is illustrated in *Figure 2*.

**Figure 2:**
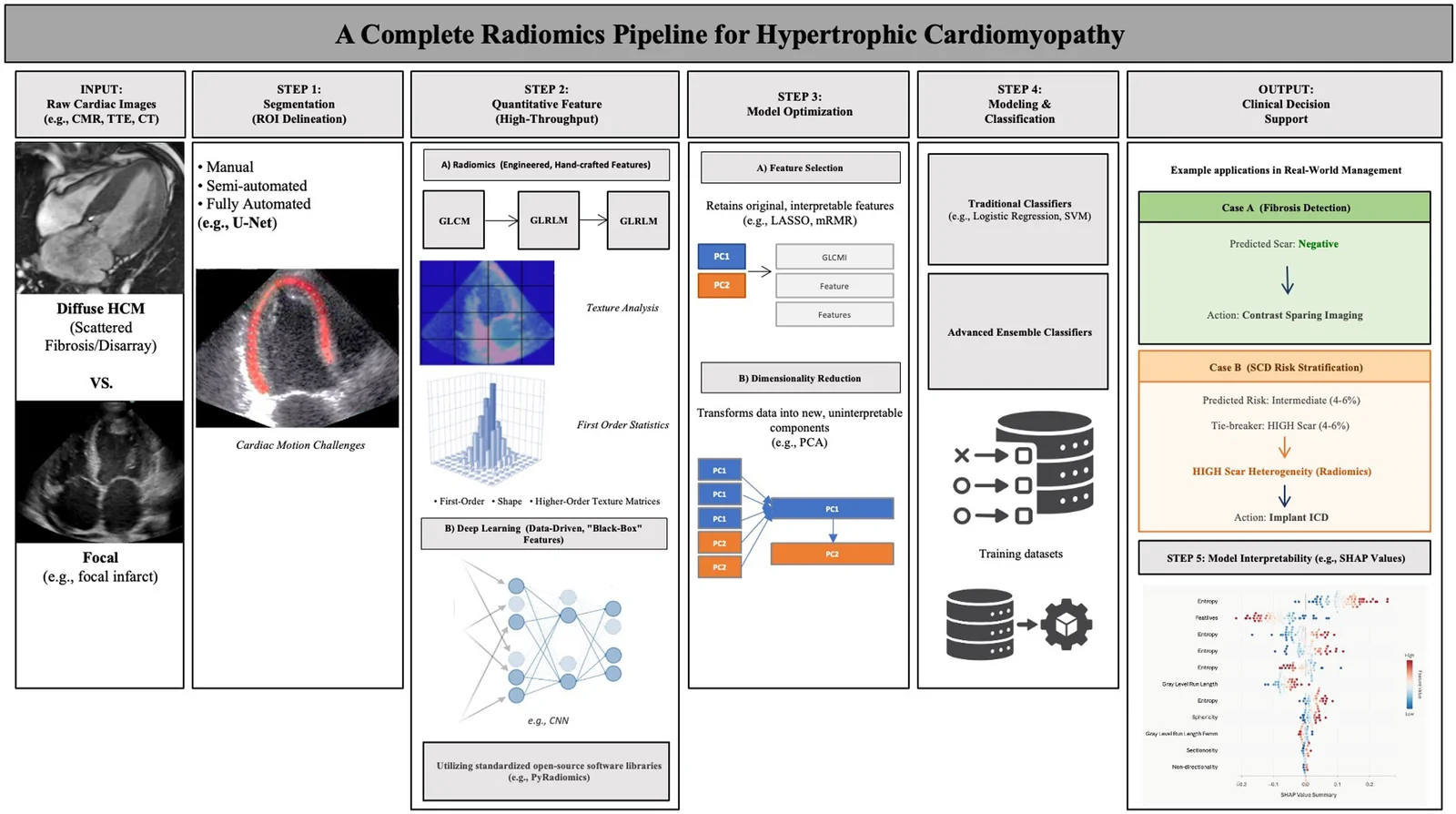
Radiomics pipeline in hypertrophic cardiomyopathy. Cardiac images (CMR, TTE or CT) are segmented to delineate the myocardial region of interest (Step 1), from which high-throughput quantitative features are extracted, either hand-crafted radiomic features or deep learning representations (Step 2). Feature selection or dimensionality reduction mitigates overfitting (Step 3) before classification with conventional or ensemble machine learning models (Step 4). Outputs may inform clinical decisions, such as contrast-sparing imaging or ICD implantation, and interpretability methods such as SHAP identify the features that drive each prediction (Step 5).

### Deep learning vs. hand-crafted radiomics

A recurring methodological question across the literature is the distinction in the diagnostic performance between workflows incorporating hand-crafted radiomic features compared to end-to-end deep learning. Hand-crafted radiomics is derived from a predefined library of mathematical descriptors that can be traced to a specific first-order, shape, or higher-order textural feature. When these are paired with subsequent statistical methods of selection, the contribution of individual features to a given prediction output can be interrogated and retraced to known pathophysiological mechanisms. However, deep learning, by contrast, learns hierarchical representations directly from the image, frequently achieving superior discrimination at the cost of interpretability, as the resulting features map less closely to explainable concepts and underlying mechanisms of disease.^50,51^ Studies have evaluated these studies against each other as well as combining them across the range of applications outlined in this review.

## Phenotypic differentiation of left ventricular hypertrophy

Differentiation of HCM from phenocopies such as hypertensive heart disease (HHD), cardiac amyloidosis (CA), and other cardiomyopathies remains a clinical problem,^52^ with misclassification rates of up to one-third reported in cohort studies.^53,54^ Furthermore, in older adults, aortic stenosis represents a common further phenocopy that must be excluded. CMR and transthoracic echocardiography (TTE) reveal hypertrophy and fibrosis but remain limited by substantial phenotypic overlap in gross imaging findings.^55^ Even global T1 mapping from CMR images to quantify diffuse interstitial fibrosis offers modest discriminatory ability^28^ compared to radiomics models, which quantify myocardial texture and shape (*Table 1*).

### Differentiating HCM from HHD

HCM and HHD both feature cardiomyocyte hypertrophy and interstitial fibrosis, but only HCM demonstrates myofibrillar disarray and patchy replacement fibrosis.^56^ These microstructural differences are difficult to resolve with standard imaging. Misclassification has important consequences. Underrecognition of HCM delays family screening and SCD risk mitigation, whereas mislabelling HHD as HCM burdens the patient with unnecessary evaluation of a non-inherited condition.^57^

### Echocardiography

Echocardiography (TTE), the first-line investigation for left ventricular hypertrophy (LVH), is limited by operator dependence and poor tissue characterization.^58^ Radiomics mitigates this by capturing myocardial textural differences, thereby improving aetiological classification without requiring additional imaging.

In a pilot study of four-chamber apical TTE, Yu *et al*.^26^ identified the interventricular septum (IVS) as a region of interest (ROI) and applied a Support Vector Machine (SVM) classifier to differentiate HCM, HHD, and uraemic cardiomyopathy. Six of 77 features proved significant (*P* < 0.05) and reproducible (ICC > 0.50). HCM exhibited the most homogeneous texture, with EtBrt, Std, and CoV yielding AUCs up to 0.87 and accuracies of 83–84%. Advancing to cine loops with automated ResUNet-based IVS segmentation, Xu *et al*.^22^ extracted temporal and frequency features. Their Boltzmann and SVM models achieved AUCs of 0.84 ± 0.05 (HHD vs. HCM) and 0.87 ± 0.04 (HCM vs. UCM), alongside >82% accuracy and up to 91% specificity, demonstrating the viability of automated pipelines.

In the largest multi-centre study to date (*n* = 1486), Moon *et al*.^16^ deployed an AI-automated LightGBM model, achieving 96% overall accuracy and AUCs of 0.96 (HCM), 0.89 (cardiac amyloidosis [CA]), and 0.86 (HHD), outperforming conventional parameter-based logistic regression. Dominant predictive features included harmonization-driven textural heterogeneity and myocardial thickness (HCM), run-length non-uniformity (CA), and shape (HHD). Although external validation was limited to a single centre and lacked cross-modality comparisons, these findings establish echocardiographic radiomics as a robust, low-cost diagnostic tool.

### Cardiac MRI

Neisius *et al*. evaluated native T1 radiomics in patients with HCM, HHD, and control patients compared with mean T1 values alone.^28^ In 232 patients (53 HHD, 108 HCM, and 71 controls) scanned with free-breathing native T1 mapping CMR at 1.5T, the entire LV myocardium was segmented manually by one primary reader and repeated by two others to select over 200 texture features. The final model achieved a diagnostic accuracy of 80.0% (c-statistic: 0.89; 95% CI: 0.77 to 1.00) in the validation set, outperforming global T1 mapping with a modest accuracy of 64% (c-statistic: 0.549; 95% CI: 0.452 to 0.640). Although this study was limited by its single-centre approach and manual segmentation, it provided early proof-of-concept evidence that T1 radiomics captures disease-specific textural patterns.^59,60^

Zhang *et al*. combined cine and LGE radiomics in a large cohort of 421 HCM and 200 HHD patients. Semi-automated segmentation using DL-based commercial software (cvi42 version 5.11.2) was applied to short-axis cine and LGE images from 3T CMR.^17^ PyRadiomics was used to extract a total of 1409 initial features with iterative selection identifying 20 optimal features by multivariate logistic regression. Logistic regression models incorporated clinical findings, cine and LGE-derived radiomics scores (radscores), and conventional imaging findings, achieving an AUC of 0.98 (CI: 0.97–0.99) in a training group and 0.98 (0.97–1.00) in a validation group, significantly outperforming models using CMR findings alone. The need for labour-intensive LGE segmentation was highlighted as a primary barrier to clinical translation.

Conversely, another trial by Liu *et al*.^19^ considered whether ROIs beyond the myocardium, such as the papillary muscle, should be considered for differentiation. Utilizing cine CMR at 3T, end-diastolic images were analysed to derive two distinct ROIs, the myocardium (MYO) and the papillary muscles (PM), for two tasks: detection of LVH and differentiation between HCM and HHD. Following redundancy reduction and feature selection with an SVM classifier, six features were used: gradient gray-level co-occurrence matrix (GLCM) correlation, original shape sphericity, original shape elongation, wavelet-LHL GLCM Imc1, log-sigma-5-0-mm-3D glszm, ZoneEntropy, and wavelet-LHH GLCM MCC. For detection of LVH, the model developed with the MYO features achieved the highest AUC (0.966 vs. 0.944 (MYO + PM) and 0.772 (PM), *P* = 0.924 and *P* < 0.001, respectively); but precision and recall were slightly higher in the MYO + PM group. However, for differentiation of LVH aetiology, the MYO + PM model achieved an AUC of 0.94, significantly outperforming conventional CMR (*P* = 0.002) and radiomics with the myocardium alone (AUC 0.91).

Building on these advances, Wang *et al*.^38^ shift the methodological focus from hand-crafted radiomic features to deep learning (ResNet-32) with native T1 maps. A CNN using a myocardial-ring ROI achieved the best performance (AUC 0.83, 95% CI: 0.70–0.96), surpassing global T1 (AUC 0.55; CI: 0.35–0.74) and radiomics classifiers (AUC 0.80; 95% CI: 0.66–0.94), remaining among the few studies that compare deep learning to radiomics.

### HCM vs. cardiac amyloidosis

CA is another key phenocopy, which can present with similar imaging findings of concentric LVH. Moon *et al*. recently demonstrated the feasibility of echocardiography radiomics for the differentiation of this condition,^16^ but CMR radiomics has equally shown promise.

In a 216-patient cohort study (50 HCM, 52 CA, 68 aortic stenosis, 15 HHD, 31 controls), a histogram-based technique was applied to cine CMR mid–short axis frames.^29^ Reproducibility remained high in this study with an ICC of 0.9, and comparisons revealed that kurtosis and skewness were key identifiers for CA, due to underlying myocardial infiltration. Whereas entropy best represented HCM due to a heterogeneous distribution of myocardial disarray. Building on these results, Jiang *et al*.^20^ studied a larger cohort of 251 patients (82 HCM, 85 CA, 84 controls) with 3.0T cine MRI. The key improvement was the use of a high-dimensional radiomic pipeline integrated with machine learning classifiers. A total of 275 features were extracted from each ROI, defined as the LV myocardium and manually segmented by two radiologists. After LASSO was applied to prevent overfitting, nineteen features were selected for analysis. The best-performing model, with radiomics and CMR, achieved a superior discriminatory performance (AUC = 0.89) over the CMR findings alone (AUC = 0.79). Again, in this study, SVM stood out as the best classifier, and run-length non-uniformity (GLevNonU) emerged as a powerful texture-based discriminator.

These studies converge on the insight that cine radiomics can capture subtle histological differences between the two conditions. Clinically, aside from the feasibility of cine MRI as a contrast-free diagnostic tool, the microstructural insight offered by radiomics enables CMR to highlight the possibility of these techniques acting as a surrogate ‘virtual biopsy’ to visualize the signature patterns of disease at a tissue level.

### Classification of other cardiomyopathies

A 118-patient study by Izquierdo *et al*. further sought to distinguish HCM from other cardiomyopathies (HCM = 25, DCM = 37, LV non-compaction = 35, and healthy = 21), whilst providing valuable context for refining use cases for quantitative imaging methods.^23^ All participants were scanned on 1.5T Siemens systems with a standard short-axis cine stack. In total, 420 radiomics features were extracted from the LV myocardium and LV cavity at end diastole (ED) and end systole (ES). A machine learning approach included classifiers such as one-vs.-rest SVMs, multinomial logistic regression, and random forest, yielding the best-performing model (random forest), which reached an overall AUC of 0.96 and a one-vs.-rest AUC of 0.95 for HCM. Remarkably, in this study, the performance of the radiomics model was similar and statistically insignificant when compared to standard CMR indices for differentiation, yet the authors maintain the relevance of their model as it achieved considerable AUC without the effort of manual delineation of the trabeculae, simplifying segmentation to the LV cavity and compact myocardium only. However, this equivalent result may be an intrinsic fallacy of the quantitative models when differentiating morphologically distinct diseases. For example, when the gross morphological changes between the asymmetric LV hypertrophy in HCM compared to the dilatation and non-compaction in DCM are already evident, the macroscopic images may already capture the majority of differences between the aetiologies, leaving little residual variance for intricate textural features to extricate. This raises the issue of high-dimensionality methods offering liability rather than added value in such situations where classifiers risk modelling scanner-specific noise or artefacts in place of biologically meaningful differences, discussed in the literature as ‘the curse of dimensionality’.^44,45^

Larger datasets have optimized the use of radiomics algorithms in cardiomyopathy differentiation. Zhang *et al*. analysed 238 subjects (HCM = 48, dilated cardiomyopathy (DCM) = 52, healthy controls = 123).^18^ Imaging with standard short-axis cine CMR, comprising ED and ES frames, was used for feature extraction. ROIs comprised the LV cavity, RV cavity, and LV myocardium. A 9-feature model achieved 91.2% accuracy with per-class AUCs of 0.94 (healthy controls), 0.97 (DCM), and 0.94 (HCM). Notably, this study also found that features derived from the right ventricle contributed sparingly to the distinction between DCM and HCM, with myocardial and LV cavity features dominating the radiomic signature, identifying specific ROIs offering valuable information beyond macroscopic indices. Despite a multi-centre approach, the authors highlighted limitations such as reliance on pre-provided contours in public datasets for ROI delineation, preventing the calculation of intraobserver variability, and a lack of patient-based features such as age, gender, or weight, restricting further analysis. Overall, these findings encourage the consideration of an optimized diagnostic workflow for patients with LVH incorporating radiomics to determine the true underlying etiology (*Figure 3*).

**Figure 3:**
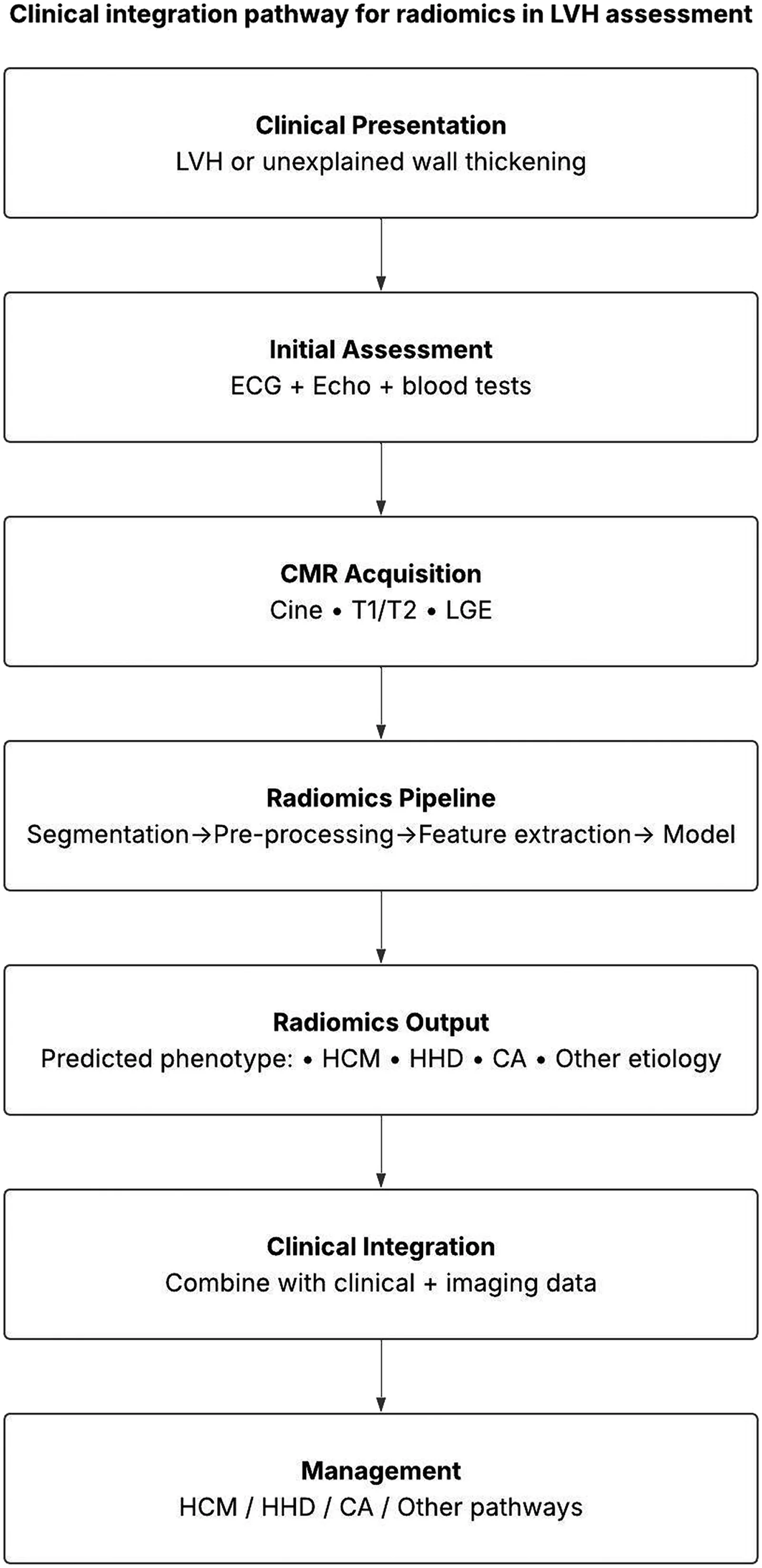
Clinical integration pathway for radiomics in left ventricular hypertrophy (LVH) assessment. This figure illustrates a proposed workflow for incorporating radiomics into the diagnostic evaluation of patients with left ventricular hypertrophy or unexplained myocardial wall thickening. Following initial clinical assessment with electrocardiography, transthoracic echocardiography, and routine blood tests, patients undergo comprehensive cardiac magnetic resonance (CMR) imaging, including cine sequences, T1/T2 mapping, and late gadolinium enhancement (LGE). A radiomics pipeline can then be applied to generate a predicted myocardial phenotype, such as hypertrophic cardiomyopathy (HCM), hypertensive heart disease (HHD), cardiac amyloidosis (CA), or other aetiologies. These outputs are integrated with conventional clinical and imaging findings to support refined diagnosis and guide downstream, disease-specific management pathways.

## Detection of fibrosis and gadolinium-sparing imaging

Myocardial fibrosis is a central component of the arrhythmogenic risk in HCM, quantified by LGE-CMR. Nearly 50% of patients with HCM are LGE-negative,^61^ but international guidelines suggest 2–5 yearly contrast-enhanced CMR scans for SCD risk stratification as a class IIb recommendation.^7,12^ Safety concerns have been raised with gadolinium, such as the risk of nephrogenic systemic fibrosis in patients with renal dysfunction, deposition in tissues such as the brain with repeated exposure.^62^ Whilst the insights offered by LGE are unparalleled, studies have investigated whether radiomics can identify fibrosis without contrast, enabling safer imaging strategies (*Table 2*).

### Cine MRI as a surrogate for LGE

Cine CMR is widely available and may contain tissue-level information detectable through radiomics. Mancio *et al*. were among the first to demonstrate this, applying machine learning to develop a model from a large multi-center dataset, externally validated on a second cohort.^32^ Images were automatically segmented to delineate LV endocardial and epicardial borders (cvi42V R DL method) with manual adjustment by an expert cardiologist. Following feature extraction, dimensionality reduction and classification (XGBoost), a seven-feature model was tested, yielding an AUC of 0.83 (95% CI: 0.77–0.89) with a sensitivity of 91% and specificity of 62%.

In a comprehensive head-to-head study, Fahmy *et al*.^30^ compared radiomics, deep learning, and hybrid models in a multi-centre cohort of 759 patients scanned with 1.5T MRI. This study also employed automatic segmentation of the LV (cvi42 image analysis package) with manual validation. Using the Pyradiomics feature extraction library, a total of 944 radiomic features were extracted from each image, with LASSO identifying the ten best performers. A deep learning model comprising convolutional neural networks (CNN) and a fully connected network (FCN) was trained end-to-end and combined or compared with the radiomics approach. The combined deep learning and radiomics model achieved the highest AUC (0.83 vs. 0.77 (deep learning), *P* = 0.006 and 0.78 (radiomics), *P* = 0.05). Furthermore, it correctly identified 43% and 28% of patients without scar in the internal and external datasets.

Recently, Pu *et al*.^31^ refined the approach by trialling LV-wide cine radiomics. 273 HCM patients scanned with 1.5T CMR were included in training and test sets at a ratio of 7:3. One radiologist manually segmented LV myocardium with assessment of interclass variability by an independent expert. Slices of the LV myocardium and maximum wall thickness (MWT) were considered, with 1688 and 1130 features extracted from the entire LV myocardium and MWT slice, respectively. Following dimensionality reduction with the Boruta algorithm and machine learning-aided classification (XGBoost), three features were selected from the MWT slice and five from the LV myocardium. Results showed that the integrated model combining conventional CMR parameters and radiomic features performed the best for detecting fibrosis (AUC of 0.90, accuracy 89.02%, sensitivity 92.54%). Despite the retrospective design and manual segmentation, this established that considering the whole LV myocardium is more reliable than slice-based models.

### Native T1 radiomics to identify scar-negative patients

While cine radiomics leverages motion and intensity patterns, native T1 mapping evaluates a direct tissue signal. Neisius *et al*.^33^ investigated whether radiomic texture analysis of native T1 maps could detect scar-specific heterogeneity invisible to mean T1 values, hence identifying patients with a low probability of scar in whom gadolinium could be avoided. Using native T1 mapping at 1.5T, the scans of 188 HCM patients were segmented to delineate the LV myocardium with LGE-positive areas labelled for comparison. Four texture descriptors, namely, histogram, run-length, co-occurrence, and local binary patterns, were extracted, and selection revealed five features with the best discriminatory capacity for LGE, which were entered into a decision-tree ensemble classifier. This model achieved a considerable c-statistic of 0.75 in cross-validation and 0.74 in an independent cohort, but most crucially, it was able to correctly identify 100% of LGE-positive patients and classified 37% of LGE-negative patients as scar-free. Avoiding contrast in up to one-third of patients whilst ensuring no scar-positive patients are missed, ascertains T1 as a powerful triage tool.

### Fibrosis assessment beyond CMR

Qin *et al*. investigated CT as an alternative to CMR by retrospectively enrolling 161 HCM patients who underwent coronary computed tomography angiography (CCTA) and CMR scanning.^34^ The LV myocardium was segmented according to the AHA 17-segment models, with each ROI yielding 1591 features. After excluding features with poor reproducibility and mitigating overfitting, CNNs and random forests were compared to develop models using combinations of radiomic signatures, clinical features, and echocardiography data. The results showed that the radiomic model achieved an AUC of 0.78 in external testing for the detection of LGE-positive segments, whereas the integrated model, comprising clinical and TTE findings as well, achieved great discrimination for extensive fibrosis (LGE/LV mass ≥ 15%) with an AUC of 0.92. The authors noted SumEntropy, Maximum (wavelet), and ClusterProminence as the top three predictive features capturing the heterogeneity of CT pixel values.

Echocardiography has also been studied for detecting fibrosis. Apical five-chamber views can be studied to train deep convolutional neural networks (DCNN) to classify the presence of LGE. In one study, a logistic regression model built from clinical and echocardiography parameters was integrated with the DCNN-derived possibility of fibrosis.^63^ This integrated model achieved an AUC of 0.86, with the authors including decision curve analysis to confirm the net clinical benefit of their model.

### SCD risk prediction and prognostic modelling

Considering that SCD remains the most feared complication of HCM, advanced risk stratification strategies remain in continuous development. Whilst current guideline-recommended risk calculators rely on limited imaging metrics with poor sensitivity, radiomics holds the potential to quantify the heterogeneity of scar in LGE-positive areas and simultaneously evaluate microstructural textural patterns, providing more sensitive biomarkers for integration into prognostic models (*Table 3*).

Wang *et al*. showed that radiomic texture features from LGE images independently predicted SCD events in 379 patients over long-term follow-up over 29 months even after adjusting for extensive LGE burden (≥15%).^24^ 11/157 features were identified as significant predictors, with local binary patterns (LBP-19) and Moment (1) emerging as the strongest, with a hazard ratio of 1.21 and 1.08, respectively. Patients with high values (LBP ≥ 4.277, Moment ≥ 4.262) experienced markedly higher rates of SCD events, identifying individuals who would otherwise have been overlooked and proving that scar heterogeneity and not just extent must be considered in risk.

Fahmy *et al*. expand this observation to a multi-centre cohort, extracting features from 1229 HCM patients’ LV LGE images.^37^ 1888 radiomic features were identified, undergoing principal component analysis to shortlist the top 3 components (PrinRads), maintaining a ratio of 1 predictor to every 10 SCD events in the data set. In their Cox regression model, PrinRads remained an independent predictor of ICD therapies and arrhythmic events, achieving a higher c-statistic (0.69) than the ACC/AHA (0.67, *P* = 0.75), and the ESC risk models (0.57, *P* = 0.02) over a follow-up period of 49 ± 28 months. Combined models incorporating the parameters of either the ESC or ACC/AHA model and radiomics were further developed, with the PrinRads-ESC model demonstrating a net reclassification index of (0.25 [95% CI: 0.08–0.43]; *P* = 0.005). This study further highlighted LGE heterogeneity as a significant predictor of SCD risk (HR: 0.07 [95% CI: 0.01–0.75]; *P* = 0.03). The authors noted that decision curve analysis revealed a net clinical benefit from this approach, especially in the intermediate-risk population for whom management is the most uncertain.

Smaller studies, such as Alis *et al*.^64^ investigated the link between texture analysis of LGE images and ventricular tachyarrhythmias. In 64 patients, also undergoing Holter monitoring, texture features were extracted from LGE-positive regions. After dimensionality reduction and machine learning classifiers, they achieved 94.1% accuracy. Durmaz *et al*.^35^ combined cine and LGE radiomics in 60 patients, of whom 17 developed sustained ventricular tachyarrhythmias. More than 1600 radiomics features were identified, and after LASSO selection and TabPFN classifier, the model achieved an AUC of 0.88. Run-length non-uniformity and wavelet-GLCM features, both reflecting textural heterogeneities, were identified as the main contributors. The translational promise remains clear that radiomics may identify and mitigate SCD risk in those patients invisible to current algorithms.

Beyond SCD, patients with HCM can experience disease progression and complications of heart failure. One model developed from a 516-patient cohort from cine CMR images found that the radiomics score served as the strongest predictor for the development of HF events in univariable and multivariable analysis. A combined model, with clinical and conventional CMR parameters, achieved a 3-year AUC of 0.77 in a validation set.^36^ Radiomics may therefore enable identification of patients with at-risk features for progressive ventricular remodelling and other major complications.

### Challenges and future perspectives

Despite encouraging results, translation of radiomics into clinical workflows remains limited by methodological and practical challenges.

### Clinical utility and incremental value

Although the majority of radiomics studies in HCM report high discrimination, often yielding AUCs >0.85, AUC alone is insufficient to justify clinical integration. Clinical decisions in HCM, such as ICD implantation, rely on absolute risk thresholds, making calibration and accurate probability estimation essential. Currently, few studies report calibration metrics or decision curve analyses to demonstrate a net clinical benefit over standard-of-care. Without these analyses, it remains unclear whether a radiomics model simply reiterates information already captured by conventional parameters. A few studies nevertheless provide a template for how future imaging biomarkers should be evaluated. Fahmy *et al*., in their sudden cardiac death model,^37^ and Akita *et al*., in echocardiographic fibrosis detection,^63^ both incorporated decision curve analysis to demonstrate net clinical benefit beyond discrimination alone, whilst Qin *et al*. reported calibration for their integrated CT model.^34^ These studies are highlighted as the current methodological benchmark, as a quantitative imaging tool intended to influence significant decisions such as ICD implantation must offer calibration and positive net benefit across clinically relevant probability thresholds. Routine integration of these analyses and a recommendation from contemporary prediction-model reporting guidelines for the inclusion of these metrics would increase the credibility of these tools for clinical adoption.

### Bias, overfitting, and generalizability

An appraisal of the current HCM radiomics literature reveals significant vulnerabilities regarding study quality and risk of bias. The most pervasive limitation is the reliance on retrospective, single-centre datasets with small sample sizes. In radiomics, the number of extracted features frequently exceeds the number of adverse clinical events, creating a high risk of overfitting. In such scenarios, machine learning classifiers may simply memorize scanner-specific noise or local acquisition protocols rather than identifying true biological signals. Consequently, while internal cross-validation often yields high discrimination, these models frequently fail when applied to new populations. Broadening the perspective to general medical imaging guidelines (such as CLEAR and CLAIM), the absolute requisite for clinical translation is independent, external validation. Future prospective studies must ensure adequately powered cohorts and openly report calibration metrics to prove true generalizability across diverse hospital systems and scanner vendors.

### Feature reproducibility and segmentation

Given the high feature-to-sample ratio typical of radiomics studies in HCM, robust feature selection is essential to reduce overfitting. Supervised approaches such as LASSO or mRMR are generally preferable when clinical interpretability is required, whereas unsupervised dimensionality reduction methods such as principal component analysis may obscure feature meaning and limit reproducibility. Transparent, cross-validated selection procedures with stability assessment are therefore recommended.

Similarly, reproducibility remains a significant concern as radiomics features are sensitive to acquisition and processing variability. Some studies acknowledge that the reproducibility of radiomic features with different manual segmentations was not tested.^30^ Similarly, the mapping of T1 radiomic features has been shown to demonstrate wide variability depending on imaging acquisition and preprocessing steps. Studies have reported that only 40–50% of features met acceptable reproducibility thresholds (ICC > 0.75) on repeat imaging or with alternative pre-processing.^65^ Reproducibility in cardiac radiomics is further complicated by cardiac motion and phase selection variability. This underscores the need for robust harmonization strategies, such as ComBat, to reduce scanner-related variation.

Campello *et al*. studied harmonization techniques across five centres in HCM patients vs. healthy controls, demonstrating that without harmonization,^66^ there was unprecedented heterogeneity in feature distribution. Downstream, manual segmentation, used by many studies, also confounds results, with reproducibility dependent on the experience of the operator. The highly trabeculated nature of the left ventricle, coupled with the variable geometry of the hypertrophied septum, makes manual segmentation highly subjective. Even minor deviations in contouring the endocardial or epicardial borders can drastically alter higher-order texture matrices. Positive steps to address this issue include the increased adoption of automated segmentation tools (e.g. ResUNet)^67^ and expert validation. Reproducibility testing must be embedded into the pipeline of future radiomics studies with test-retest sampling and sensitivity analysis.

### Biological interpretability

Whilst LGE reflects areas of fibrosis, higher-order texture features extracted by radiomics, such as LBP and Moment,^24^ are not currently mapped to known histopathological processes.^68^ Current literature focuses heavily on adult cohorts with classic asymmetrical septal hypertrophy, leaving significant phenotypic ‘blind spots.’ For example, Apical HCM, characterized by a ‘spade-like’ left ventricular cavity and dense apical crowding, presents distinct imaging challenges. The apex is notoriously vulnerable to near-field artefacts in echocardiography and partial volume effects in CMR. Consequently, radiomics models trained predominantly on septal hypertrophy are highly likely to misclassify or fail when applied to apical variants. Similarly, paediatric HCM is absent from current radiomic validation studies. Paediatric patients exhibit vastly different baseline heart rates, body surface areas, and progressive phenotypic penetrance compared to adults. The genetic and histological underpinnings in paediatric cohorts often manifest with different temporal imaging signatures.^27^ To be clinically viable, future radiomic algorithms must explicitly include training data from these under-represented subgroups to ensure equitable and accurate diagnostic performance.^69–71^

Furthermore, most classification models are limited by the underexplored assumption of mutually exclusive diagnoses. In practice, a considerable proportion of patients could have concurrent pathologies such as HHD and HCM in the setting of comorbid arterial hypertension or transthyretin amyloidosis in elderly patients with existing aortic stenosis.^72^ Classifiers trained to assign a diagnosis label may fail to comprehend the complexity of these mixed phenotypes. Similarly, the diagnostic accuracy of these studies is relative to the reference standard of the models. Most models are trained on a clinical or imaging-based diagnosis, which is imperfect and may fail to encompass borderline or atypical presentations such as apical HCM with a maximum wall thickness below 12 mm or grade 1 cardiac amyloidosis, which carries a significant challenge of diagnostic uncertainty from imaging.^73,74^ Validating models against these standards risks the inheritance of uncertainty with seemingly accurate result outputs reproducing the errors of current diagnostic limitation.

### Concerns with data sharing

Development of generalizable radiomics models requires large multi-center datasets, yet data sharing is limited by privacy and governance restrictions.^75,76^ Federated learning (FL) has emerged as a potential solution to mitigate the issues surrounding patient-sensitive data. In FL, a central server aggregator receives model updates instead of raw data, allowing inter-institutional data sharing with fewer concerns.^76^ Linardos *et al*. explored this concept in a simulation of FL across four centres using CMR images to classify healthy vs. HCM patients.^21^ Performances remained equivalent to a centralized approach of sharing raw data, without the risk of data leakage. A recent large cohort federated learning study using cardiac CT images further supported that these models can be implemented as a scalable solution for radiomics research.^77^

### Reporting and clinical implementation

Ultimately, clinical translation of radiomics in HCM workflows depends on transparent reporting and alignment with guideline-driven standards. Several current studies fall short on these expectations.^78^ Critical methodological details for reproducibility are inconsistently reported, while calibration and decision-curve analysis to justify clinical value are sparsely assessed. Consensus bodies have defined what constitutes good practice in radiomics studies. The Image Biomarker Standardisation Initiative (IBSI) defines feature nomenclature, as well as providing reporting guidelines and recommendations for image pre-processing.^79^ Whereas the CLEAR checklist (CheckList for EvaluAtion of Radiomics) sets a list of mandatory reporting items, from segmentation to data sharing. Furthermore, as radiomics is rapidly advancing into the field of AI, broader guidelines such as TRIPOD AI,^80^ and CLAIM^81^ emphasize the need for calibration, pre-registration, and transparent reporting. Recent AHA statements underscore the need for fairness and monitoring of AI-based cardiovascular imaging tools,^82^ paving the way for clinical decision support.

## Data Availability

No new data were generated or analysed in support of this research.

## Relationship with industry

The authors report no relationships with industry relevant to the contents of this manuscript.

## Lead author biography

Sukruth Pradeep Kundur is a final-year medical student at King’s College London. His research interests span cardiovascular medicine, including atherosclerotic heart disease, inflammatory myocardial disease, and cardiovascular imaging.

## References

[1] Massera D, Sherrid MV, Maron MS, Rowin EJ, Maron BJ. How common is hypertrophic cardiomyopathy… really?: Disease prevalence revisited 27 years after CARDIA. Int J Cardiol 2023;382:64–7. 10.1016/j.ijcard.2023.04.005 37028711

[2] Maron BJ, Haas TS, Ahluwalia A, Murphy CJ, Garberich RF. Demographics and epidemiology of sudden deaths in young competitive athletes: from the United States National Registry. Am J Med 2016;129:1170–7. 10.1016/j.amjmed.2016.02.031 27039955

[3] Miron A, Lafreniere-Roula M, Steve Fan C-P, Armstrong KR, Dragulescu A, Papaz T et al A validated model for sudden cardiac death risk prediction in pediatric hypertrophic cardiomyopathy. Circulation 2020;142:217–29. 10.1161/CIRCULATIONAHA.120.047235 PMC7365676 32418493

[4] O'Mahony C, Jichi F, Pavlou M, Monserrat L, Anastasakis A, Rapezzi C et al A novel clinical risk prediction model for sudden cardiac death in hypertrophic cardiomyopathy (HCM risk-SCD). Eur Heart J 2014;35:2010–20. 10.1093/eurheartj/eht439 24126876

[5] Authors/Task Force members; Elliott PM, Anastasakis A, Borger MA, Borggrefe M, Cecchi F et al 2014 ESC Guidelines on diagnosis and management of hypertrophic cardiomyopathy: the Task Force for the Diagnosis and Management of Hypertrophic Cardiomyopathy of the European Society of Cardiology (ESC). Eur Heart J 2014;35:2733–79. 10.1093/eurheartj/ehu284 25173338

[6] Marian AJ, Braunwald E. Hypertrophic cardiomyopathy: genetics, pathogenesis, clinical manifestations, diagnosis, and therapy. Circ Res 2017;121:749–70. 10.1161/CIRCRESAHA.117.311059 PMC5654557 28912181

[7] Ommen SR, Ho CY, Asif IM, Balaji S, Burke MA, Day SM et al 2024 AHA/ACC/AMSSM/HRS/PACES/SCMR Guideline for the management of hypertrophic cardiomyopathy: a report of the American Heart Association/American College of Cardiology Joint Committee on Clinical Practice Guidelines. Circulation 2024;149:e1239–311. 10.1161/CIR.0000000000001250 38718139

[8] Dong T, Gilliland Y, Kramer CM, Theodore A, Desai M. Multimodality imaging of hypertrophic cardiomyopathy. Prog Cardiovasc Dis 2023;80:14–24. 10.1016/j.pcad.2023.08.004 37586654

[9] Ashkir Z, Samat AHA, Ariga R, Finnigan LEM, Jermy S, Akhtar MA et al Myocardial disarray and fibrosis across hypertrophic cardiomyopathy stages associate with ECG markers of arrhythmic risk. Eur Heart J Cardiovasc Imaging 2025;26:218–28. 10.1093/ehjci/jeae260 PMC11781828 39417278

[10] Ireland CG, Ho CY. Genetic testing in hypertrophic cardiomyopathy. Am J Cardiol 2024;212S:S4–13. 10.1016/j.amjcard.2023.10.032 38368035

[11] Méndez C, Soler R, Rodríguez E, Barriales R, Ochoa JP, Monserrat L. Differential diagnosis of thickened myocardium: an illustrative MRI review. Insights Imaging 2018;9:695–707. 10.1007/s13244-018-0655-9 PMC6206373 30302634

[12] Arbelo E, Protonotarios A, Gimeno JR, Arbustini E, Barriales-Villa R, Basso C et al 2023 ESC Guidelines for the management of cardiomyopathies. Eur Heart J 2023;44:3503–626. 10.1093/eurheartj/ehad194 37622657

[13] Maron MS, Rowin EJ, Maron BJ. The paradigm of sudden death prevention in hypertrophic cardiomyopathy. Am J Cardiol 2024;212S:S64–76. 10.1016/j.amjcard.2023.10.076 38368038

[14] Abdelfattah OM, Martinez M, Sayed A, Elrefaei M, Abushouk AI, Hassan A et al Temporal and global trends of the incidence of sudden cardiac death in hypertrophic cardiomyopathy. JACC Clin Electrophysiol 2022;8:1417–27. 10.1016/j.jacep.2022.07.012 36424010

[15] Al-Mallah MH . Radiomics in hypertrophic cardiomyopathy. JACC Cardiovasc Imaging 2019;12:1955–7. 10.1016/j.jcmg.2019.02.004 30878414

[16] Moon I, Lee J, Lee S-A, Jeong D, Jeon J, Jang Y et al Artificial intelligence-enhanced analysis of echocardiography-based radiomic features for myocardial hypertrophy detection and etiology differentiation. Circ Cardiovasc Imaging 2025;18:e017436. 10.1161/CIRCIMAGING.124.017436 40143828

[17] Zhang H, Tian J, Zhang C, Wang H, Hui K, Wang T et al Discrimination models with radiomics features derived from cardiovascular magnetic resonance images for distinguishing hypertensive heart disease from hypertrophic cardiomyopathy. Cardiovasc Diagn Ther 2024;14:129–42. 10.21037/cdt-23-350 PMC10904305 38434569

[18] Zhang X, Cui C, Zhao S, Xie L, Tian Y. Cardiac magnetic resonance radiomics for disease classification. Eur Radiol 2023;33:2312–23. 10.1007/s00330-022-09236-x 36378251

[19] Liu Q, Lu Q, Chai Y, Tao Z, Wu Q, Jiang M et al Papillary-muscle-derived radiomic features for hypertrophic cardiomyopathy versus hypertensive heart disease classification. Diagnostics (Basel) 2023;13:1544. 10.3390/diagnostics13091544 PMC10177511 37174935

[20] Jiang S, Zhang L, Wang J, Li X, Hu S, Fu Y et al Differentiating between cardiac amyloidosis and hypertrophic cardiomyopathy on non-contrast cine-magnetic resonance images using machine learning-based radiomics. Front Cardiovasc Med 2022;9:1001269. 10.3389/fcvm.2022.1001269 PMC9643770 36386316

[21] Linardos A, Kushibar K, Walsh S, Gkontra P, Lekadir K. Federated learning for multi-center imaging diagnostics: a simulation study in cardiovascular disease. Sci Rep 2022;12:3551. 10.1038/s41598-022-07186-4 PMC8894335 35241683

[22] Xu Z, Yu F, Zhang B, Zhang Q. Intelligent diagnosis of left ventricular hypertrophy using transthoracic echocardiography videos. Comput Methods Programs Biomed 2022;226:107182. 10.1016/j.cmpb.2022.107182 36257197

[23] Izquierdo C, Casas G, Martin-Isla C, Campello VM, Guala A, Gkontra P et al Radiomics-based classification of left ventricular non-compaction, hypertrophic cardiomyopathy, and dilated cardiomyopathy in cardiovascular magnetic resonance. Front Cardiovasc Med 2021;8:764312. 10.3389/fcvm.2021.764312 PMC8586199 34778415

[24] Wang J, Bravo L, Zhang J, Liu W, Wan K, Sun J et al Radiomics analysis derived from LGE-MRI predict sudden cardiac death in participants with hypertrophic cardiomyopathy. Front Cardiovasc Med 2021;8:766287. 10.3389/fcvm.2021.766287 PMC8702805 34957254

[25] Antonopoulos AS, Boutsikou M, Simantiris S, Angelopoulos A, Lazaros G, Panagiotopoulos I et al Machine learning of native T1 mapping radiomics for classification of hypertrophic cardiomyopathy phenotypes. Sci Rep 2021;11:23596. 10.1038/s41598-021-02971-z PMC8654857 34880319

[26] Yu F, Huang H, Yu Q, Ma Y, Zhang Q, Zhang B. Artificial intelligence-based myocardial texture analysis in etiological differentiation of left ventricular hypertrophy. Ann Transl Med 2021;9:108. 10.21037/atm-20-4891 PMC7867873 33569410

[27] Wang J, Yang F, Liu W, Sun J, Han Y, Li D et al Radiomic analysis of native T1 mapping images discriminates between MYH7 and MYBPC3-related hypertrophic cardiomyopathy. J Magn Reson Imaging 2020;52:1714–21. 10.1002/jmri.27209 32525266

[28] Neisius U, El-Rewaidy H, Nakamori S, Rodriguez J, Manning WJ, Nezafat R. Radiomic analysis of myocardial native T1 imaging discriminates between hypertensive heart disease and hypertrophic cardiomyopathy. JACC Cardiovasc Imaging 2019;12:1946–54. 10.1016/j.jcmg.2018.11.024 PMC7032053 30660549

[29] Schofield R, Ganeshan B, Fontana M, Nasis A, Castelletti S, Rosmini S et al Texture analysis of cardiovascular magnetic resonance cine images differentiates aetiologies of left ventricular hypertrophy. Clin Radiol 2019;74:140–9. 10.1016/j.crad.2018.09.016 30527518

[30] Fahmy AS, Rowin EJ, Arafati A, Al-Otaibi T, Maron MS, Nezafat R. Radiomics and deep learning for myocardial scar screening in hypertrophic cardiomyopathy. J Cardiovasc Magn Reson 2022;24:40. 10.1186/s12968-022-00869-x PMC9235098 35761339

[31] Pu C, Hu X, Lv S, Wu Y, Yu F, Zhu W et al Identification of fibrosis in hypertrophic cardiomyopathy: a radiomic study on cardiac magnetic resonance cine imaging. Eur Radiol 2023;33:2301–11. 10.1007/s00330-022-09217-0 PMC10017609 36334102

[32] Mancio J, Pashakhanloo F, El-Rewaidy H, Jang J, Joshi G, Csecs I et al Machine learning phenotyping of scarred myocardium from cine in hypertrophic cardiomyopathy. Eur Heart J Cardiovasc Imaging 2022;23:532–42. 10.1093/ehjci/jeab056 PMC9125682 33779725

[33] Neisius U, El-Rewaidy H, Kucukseymen S, Tsao CW, Mancio J, Nakamori S et al Texture signatures of native myocardial T1 as novel imaging markers for identification of hypertrophic cardiomyopathy patients without scar. J Magn Reson Imaging 2020;52:906–19. 10.1002/jmri.27048 PMC9190206 31971296

[34] Qin L, Chen C, Gu S, Zhou M, Xu Z, Ge Y et al A radiomic approach to predict myocardial fibrosis on coronary CT angiography in hypertrophic cardiomyopathy. Int J Cardiol 2021;337:113–8. 10.1016/j.ijcard.2021.04.060 33961944

[35] Durmaz ES, Karabacak M, Ozkara BB, Kargın OA, Demir B, Raimoglou D et al Machine learning and radiomics for ventricular tachyarrhythmia prediction in hypertrophic cardiomyopathy: insights from an MRI-based analysis. Acta Radiologica 2024;65:1473–81. 10.1177/02841851241283041 39350610

[36] Zhang H, Zhao L, Wang H, Yi Y, Hui K, Zhang C et al Radiomics from cardiovascular MR cine images for identifying patients with hypertrophic cardiomyopathy at high risk for heart failure. Radiol Cardiothorac Imaging 2024;6:e230323. 10.1148/ryct.230323 PMC10912890 38385758

[37] Fahmy AS, Rowin EJ, Jaafar N, Chan RH, Rodriguez J, Nakamori S et al Radiomics of late gadolinium enhancement reveals prognostic value of myocardial scar heterogeneity in hypertrophic cardiomyopathy. JACC Cardiovasc Imaging 2024;17:16–27. 10.1016/j.jcmg.2023.05.003 37354155

[38] Wang ZC, Fan ZZ, Liu XY, Zhu MJ, Jiang SS, Tian S et al Deep learning for discrimination of hypertrophic cardiomyopathy and hypertensive heart disease on MRI native T1 maps. J Magn Reson Imaging 2024;59:837–48. 10.1002/jmri.28904 37431848

[39] Van Timmeren JE, Cester D, Tanadini-Lang S, Alkadhi H, Baessler B. Radiomics in medical imaging—“how-to” guide and critical reflection. Insights Imaging 2020;11:91. 10.1186/s13244-020-00887-2 PMC7423816 32785796

[40] Lin Y-C, Lin G, Pandey S, Yeh C-H, Wang J-J, Lin C-Y et al Fully automated segmentation and radiomics feature extraction of hypopharyngeal cancer on MRI using deep learning. Eur Radiol 2023;33:6548–56. 10.1007/s00330-023-09827-2 PMC10415433 37338554

[41] Yu X, Yao X, Wu B, Zhou H, Xia S, Su W et al Using deep learning method to identify left ventricular hypertrophy on echocardiography. Int J Cardiovasc Imaging 2022;38:759–69. 10.1007/s10554-021-02461-3 PMC11130004 34757566

[42] Nazari M, Emami H, Rabiei R, Rabiee HR, Salari A, Sadr H. Enhancing cardiac function assessment: developing and validating a domain adaptive framework for automating the segmentation of echocardiogram videos. Comput Med Imaging Graph 2025;124:102627. 10.1016/j.compmedimag.2025.102627 40834844

[43] Li S, Kendrick J, Ebert MA, Hassan GM, Barry N, Wright K et al Auto-segmentation, radiomic reproducibility, and comparison of radiomics between manual and AI-derived segmentations for coronary arteries in cardiac [^18^F]NaF PET/CT images. EJNMMI Phys 2025;12:42. 10.1186/s40658-025-00751-6 PMC12034606 40287890

[44] Berisha V, Krantsevich C, Hahn PR, Hahn S, Dasarathy G, Turaga P et al Digital medicine and the curse of dimensionality. NPJ Digit Med 2021;4:153. 10.1038/s41746-021-00521-5 PMC8553745 34711924

[45] Marzi C, Marfisi D, Barucci A, Del Meglio J, Lilli A, Vignali C et al Collinearity and dimensionality reduction in radiomics: effect of preprocessing parameters in hypertrophic cardiomyopathy magnetic resonance T1 and T2 mapping. Bioengineering (Basel) 2023;10:80. 10.3390/bioengineering10010080 PMC9854492 36671652

[46] Shoemaker K, Ger R, Court LE, Aerts H, Vannucci M, Peterson CB. Bayesian feature selection for radiomics using reliability metrics. Front Genet 2023;14:1112914. 10.3389/fgene.2023.1112914 PMC10030957 36968604

[47] Nohara Y, Matsumoto K, Soejima H, Nakashima N. Explanation of machine learning models using shapley additive explanation and application for real data in hospital. Comput Methods Programs Biomed 2022;214:106584. 10.1016/j.cmpb.2021.106584 34942412

[48] Sarvamangala DR, Kulkarni RV. Convolutional neural networks in medical image understanding: a survey. Evol Intell 2022;15:1–22. 10.1007/s12065-020-00540-3 PMC7778711 33425040

[49] Vickers AJ, Holland F. Decision curve analysis to evaluate the clinical benefit of prediction models. Spine J 2021;21:1643–8. 10.1016/j.spinee.2021.02.024 PMC8413398 33676020

[50] Rundo L, Militello C. Image biomarkers and explainable AI: handcrafted features versus deep learned features. Eur Radiol Exp 2024;8:130. 10.1186/s41747-024-00529-y PMC11576747 39560820

[51] Haupt M, Maurer MH, Thomas RP. Explainable artificial intelligence in radiological cardiovascular imaging—a systematic review. Diagnostics 2025;15:1399. 10.3390/diagnostics15111399 PMC12155260 40506971

[52] Bakalakos A, Monda E, Elliott PM. The diagnostic and therapeutic implications of phenocopies and mimics of hypertrophic cardiomyopathy. Can J Cardiol 2024;40:754–65. 10.1016/j.cjca.2024.02.025 38447917

[53] Magnusson P, Palm A, Branden E, Mörner S. Misclassification of hypertrophic cardiomyopathy: validation of diagnostic codes. Clin Epidemiol 2017;9:403–10. 10.2147/CLEP.S139300 PMC5557115 28848363

[54] Nielsen SK, Rasmussen TB, Hey TM, Zaremba T, Lassen JF, Mogensen J. Frequency of misdiagnosis in hypertrophic cardiomyopathy. Eur Heart J Qual Care Clin Outcomes 2025;11:105–12. 10.1093/ehjqcco/qcae031 38664062

[55] Zhao Q, Chen Z, Qi C, Xu S, Ren R, Li W et al Cardiac magnetic resonance imaging for discrimination of hypertensive heart disease and hypertrophic cardiomyopathy: a systematic review and meta-analysis. Front Cardiovasc Med 2024;11:1421013. 10.3389/fcvm.2024.1421013 PMC11327824 39156132

[56] Rodrigues JCL, Rohan S, Ghosh Dastidar A, Harries I, Lawton CB, Ratcliffe LE et al Hypertensive heart disease versus hypertrophic cardiomyopathy: multi-parametric cardiovascular magnetic resonance discriminators when end-diastolic wall thickness ≥ 15 mm. Eur Radiol 2017;27:1125–35. 10.1007/s00330-016-4468-2 27368925

[57] Wang A . Hypertension and hypertrophic cardiomyopathy. In: Naidu SS, ed. Hypertrophic Cardiomyopathy: Cham: Springer International Publishing; 2019. p221–30.

[58] Mandeş L, Roşca M, Ciupercă D, Popescu BA. The role of echocardiography for diagnosis and prognostic stratification in hypertrophic cardiomyopathy. J Echocardiogr 2020;18:137–48. 10.1007/s12574-020-00467-9 PMC7473965 32301048

[59] Baeßler B, Mannil M, Maintz D, Alkadhi H, Manka R. Texture analysis and machine learning of non-contrast T1-weighted MR images in patients with hypertrophic cardiomyopathy—preliminary results. Eur J Radiol 2018;102:61–7. 10.1016/j.ejrad.2018.03.013 29685546

[60] Shi RY, Wu R, An DAL, Chen BH, Wu CW, Du L et al Texture analysis applied in T1 maps and extracellular volume obtained using cardiac MRI in the diagnosis of hypertrophic cardiomyopathy and hypertensive heart disease compared with normal controls. Clin Radiol 2021;76:236.e9–e19. 10.1016/j.crad.2020.11.001 33272531

[61] Pan JA . Late gadolinium enhancement in hypertrophic cardiomyopathy. JACC Cardiovasc Imaging 2024;17:498–500. 10.1016/j.jcmg.2023.11.013 PMC11227108 38180415

[62] Gulani V, Calamante F, Shellock FG, Kanal E, Reeder SB. Gadolinium deposition in the brain: summary of evidence and recommendations. Lancet Neurol 2017;16:564–70. 10.1016/S1474-4422(17)30158-8 28653648

[63] Akita K, Kusunose K, Haga A, Shimomura T, Kosaka Y, Ishiyama K et al Deep learning of echocardiography distinguishes between presence and absence of late gadolinium enhancement on cardiac magnetic resonance in patients with hypertrophic cardiomyopathy. Echo Res Pract 2024;11:23. 10.1186/s44156-024-00059-8 PMC11472433 39396969

[64] Alis D, Guler A, Yergin M, Asmakutlu O. Assessment of ventricular tachyarrhythmia in patients with hypertrophic cardiomyopathy with machine learning-based texture analysis of late gadolinium enhancement cardiac MRI. Diagn Interv Imaging 2020;101:137–46. 10.1016/j.diii.2019.10.005 31727603

[65] Marfisi D, Tessa C, Marzi C, Del Meglio J, Linsalata S, Borgheresi R et al Image resampling and discretization effect on the estimate of myocardial radiomic features from T1 and T2 mapping in hypertrophic cardiomyopathy. Sci Rep 2022;12:10186. 10.1038/s41598-022-13937-0 PMC9205876 35715531

[66] Campello VM, Martín-Isla C, Izquierdo C, Guala A, Palomares JFR, Viladés D et al Minimising multi-centre radiomics variability through image normalisation: a pilot study. Sci Rep 2022;12:12532. 10.1038/s41598-022-16375-0 PMC9307565 35869125

[67] Majumder S, Katz S, Kontos D, Roshkovan L. State of the art: radiomics and radiomics-related artificial intelligence on the road to clinical translation. BJR Open 2024;6:tzad004. 10.1093/bjro/tzad004 PMC10860524 38352179

[68] Tomaszewski MR, Gillies RJ. The biological meaning of radiomic features. Radiology 2021;298:505–16. 10.1148/radiol.2021202553 PMC7924519 33399513

[69] Babu M, Snyder M. Multi-omics profiling for health. Mol Cell Proteomics 2023;22:100561. 10.1016/j.mcpro.2023.100561 PMC10220275 37119971

[70] Garmany R, Bos JM, Tester DJ, Giudicessi JR, Dos Remedios CG, Dasari S et al Multi-omic architecture of obstructive hypertrophic cardiomyopathy. Circ Genom Precis Med 2023;16:e003756. 10.1161/CIRCGEN.122.003756 36802768

[71] Luo Y, Zhao C, Chen F. Multiomics research: principles and challenges in integrated analysis. Biodes Res 2024;6:0059. 10.34133/bdr.0059 PMC11844812 39990095

[72] Scully PR, Patel KP, Treibel TA, Thornton GD, Hughes RK, Chadalavada S et al Prevalence and outcome of dual aortic stenosis and cardiac amyloid pathology in patients referred for transcatheter aortic valve implantation. Eur Heart J 2020;41:2759–67. 10.1093/eurheartj/ehaa170 PMC7395329 32267922

[73] Hughes RK, Shiwani H, Rosmini S, Augusto JB, Burke L, Jiang Y et al Improved diagnostic criteria for apical hypertrophic cardiomyopathy. JACC Cardiovasc Imaging 2024;17:501–12. 10.1016/j.jcmg.2023.07.012 37831014

[74] Gillmore JD, Maurer MS, Falk RH, Merlini G, Damy T, Dispenzieri A et al Nonbiopsy diagnosis of cardiac transthyretin amyloidosis. Circulation 2016;133:2404–12. 10.1161/CIRCULATIONAHA.116.021612 27143678

[75] Miles K . Radiomics for personalised medicine: the long road ahead. Br J Cancer 2020;122:929–30. 10.1038/s41416-019-0699-8 PMC7109132 31937924

[76] Rehman MHU, Hugo Lopez Pinaya W, Nachev P, Teo JT, Ourselin S, Cardoso MJ. Federated learning for medical imaging radiology. Br J Radiol 2023;96:20220890. 10.1259/bjr.20220890 PMC10546441 38011227

[77] Tölle M, Garthe P, Scherer C, Seliger JM, Leha A, Krüger N et al Real world federated learning with a knowledge distilled transformer for cardiac CT imaging. NPJ Digit Med 2025;8:88. 10.1038/s41746-025-01434-3 PMC11802793 39915633

[78] Kocak B, Ponsiglione A, Stanzione A, Ugga L, Klontzas ME, Cannella R et al CLEAR guideline for radiomics: early insights into current reporting practices endorsed by EuSoMII. Eur J Radiol 2024;181:111788. 10.1016/j.ejrad.2024.111788 39437630

[79] Zwanenburg A, Vallières M, Abdalah MA, Aerts HJWL, Andrearczyk V, Apte A et al The image biomarker standardization initiative: standardized quantitative radiomics for high-throughput image-based phenotyping. Radiology 2020;295:328–38. 10.1148/radiol.2020191145 PMC7193906 32154773

[80] Collins GS, Moons KGM, Dhiman P, Riley RD, Beam AL, Van Calster B et al TRIPOD+AI statement: updated guidance for reporting clinical prediction models that use regression or machine learning methods. BMJ 2024;385:e078378. 10.1136/bmj-2023-078378 PMC11019967 38626948

[81] Tejani AS, Klontzas ME, Gatti AA, Mongan JT, Moy L, Park SH et al Checklist for artificial intelligence in medical imaging (CLAIM): 2024 update. Radiol Artif Intell 2024;6:e240300. 10.1148/ryai.240300 PMC11304031 38809149

[82] Hanneman K, Playford D, Dey D, Van Assen M, Mastrodicasa D, Cook TS et al Value creation through artificial intelligence and cardiovascular imaging: a scientific statement from the American Heart Association. Circulation 2024;149:e296–311. 10.1161/CIR.0000000000001202 38193315

